# Does the beta regularization parameter of bayesian penalized likelihood reconstruction always affect the quantification accuracy and image quality of positron emission tomography computed tomography?

**DOI:** 10.1002/acm2.13129

**Published:** 2021-03-08

**Authors:** Zhifang Wu, Binwei Guo, Bin Huang, Bin Zhao, Zhixing Qin, Xinzhong Hao, Meng Liang, Jun Xie, Sijin Li

**Affiliations:** ^1^ Department of Nuclear Medicine First Hospital of Shanxi Medical University Taiyuan Shanxi P.R. China; ^2^ Molecular Imaging Precision Medical Collaborative Innovation Center Shanxi Medical University Taiyuan Shanxi P.R. China; ^3^ Department of Biochemistry and Molecular Biology Shanxi Medical University Taiyuan Shanxi P.R. China

**Keywords:** Bayesian penalized likelihood, PET/CT, small pulmonary nodules

## Abstract

**Purpose:**

This study aims to provide a detailed investigation on the noise penalization factor in Bayesian penalized likelihood (BPL)‐based algorithm, with the utilization of partial volume effect correction (PVC), so as to offer the suitable beta value and optimum standardized uptake value (SUV) parameters in clinical practice for small pulmonary nodules.

**Methods:**

A National Electrical Manufacturers Association (NEMA) image‐quality phantom was scanned and images were reconstructed using BPL with beta values ranged from 100 to 1000. The recovery coefficient (RC), contrast recovery (CR), and background variability (BV) were measured to assess the quantification accuracy and image quality. In the clinical assessment, lesions were categorized into sub‐centimeter (<10 mm, n = 7) group and medium size (10–30 mm, n = 16) group. Signal‐to‐noise ratio (SNR) and contrast‐to‐noise ratio (CNR) were measured to evaluate the image quality and lesion detectability. With PVC was performed, the impact of beta values on SUVs (SUVmax, SUVmean, SUVpeak) of small pulmonary nodules was evaluated. Subjective image analysis was performed by two experienced readers.

**Results:**

With the increasing of beta values, RC, CR, and BV decreased gradually in the phantom work. In the clinical study, SNR and CNR of both groups increased with the beta values (*P* < 0.001), although the sub‐centimeter group showed increases after the beta value reached over 700. In addition, highly significant negative correlations were observed between SUVs and beta values for both lesion‐size groups before the PVC (*P* < 0.001 for all). After the PVC, SUVpeak measured from the sub‐centimeter group was no significantly different among different beta values (*P* = 0.830).

**Conclusion:**

Our study suggests using SUVpeak as the quantification parameter with PVC performed to mitigate the effects of beta regularization. Beta values between 300 and 400 were preferred for pulmonary nodules smaller than 30 mm.

## 1 INTRODUCTION

Positron emission tomography (PET) is used extensively in clinical oncology for tumor detection, staging and therapy response assessment. It also facilitates theranostic PET‐guided therapy protocols.^1^ A range of image‐derived numerical metrics, such as the standardized uptake value (SUV) or the kinetic attributes are used in PET for quantitative analysis, which is better than visual assessment for distinguishing effective early treatment response from ineffective one in oncotherapy.^2^ Recently, progress and innovative developments of new probes targeting different biological features (cell proliferation, amino acid transport/metabolism, integrin receptor expression in angiogenesis and metastasis),^3^ as well as the use of artificial intelligence‐based techniques (machine learning and deep learning of radiomics from PET imaging),^4^ are revolutionizing clinical practice in oncology. As a result, the quantitative accuracy is extremely important when quantitative PET is still challenged by several degrading physical factors related to the physics of PET imaging. The quantification accuracy is inherently compromised by the limited spatial resolution due to partial volume effect (PVE), which results in the underestimation of radiotracer uptake, particularly for lesions smaller than 2 times of the system spatial resolution,^5^ such as sub‐centimeter pulmonary nodules and lymph nodes PVEs include both spill‐in and spill‐out of activity to and from a region‐ or organ‐of‐interest. Activities from the hot regions may interfere with PET quantification and visualization of nearby lesions, resulting in an overestimation of their SUVs. This effect is often referred to as the “spill‐in.”^6^ On the opposite, activities are usually underestimated due to the “spill‐out” of counts to neighboring regions with lower activity. For example, in dynamic cardiac images, activities in ventricular chamber are usually underestimated due to the spill‐out effect, and overestimation of activity in neighboring regions such as the blood pool is caused by spill‐in effect.^7^ These errors in the estimated activities can affect quantitative parameters. Therefore, compensating for PVEs should be carefully performed to ensure the accuracy of PET measurements. One of the simple correction methods to overcome the bias caused by PVE is to use the recover coefficient (ratio of observed to true activity, RC).^8^ When applying this method, the measured lesion uptake in a ROI is divided by a correction factor RC. Srinivas et al. demonstrated a study on performing PVE correction (PVC) by using RC values. They firstly derived RC values from a NEMA phantom with six spheres at different spheres to background ratios (8:1, 6:1, and 4:1), and then a lookup table was generated. This lookup table was plotted with RC versus sphere/background activity ratio for spheres of different sizes.^9^ The PVE‐corrected standardized uptake value (SUV) of lesions investigated in their study were obtained by plugging the original SUV into the equation derived from the lookup table. This PVC method using RC values is classified as a “regional correction” method, which means it does not yield a PVE‐corrected image but only corrects the bias in an ROI and obtains a PVE‐corrected uptake value.^8^ As a crucial aspect, the PET reconstruction algorithm has a huge impact on the accuracy of SUV measurement.^10^, ^11^, ^12^ Currently, the most widely used reconstruction algorithm in clinical practice is ordered subsets expectation maximization (OSEM). OSEM is not able to reach full convergence because the noise in the image grows with each iteration and hence there exists a compromise between iteration and noise resulting in partial convergence^13^. Besides, postsmoothing method for noise suppression^14^ improves the acceptance of image quality but at a cost of reduced quantitative accuracy and volume distortions in small objects.

A Bayesian penalized likelihood (BPL)‐based reconstruction algorithm (Q.Clear, GE Healthcare, Milwaukee) has been recently introduced for clinical routine with distinct advantages over OSEM. The BPL‐based algorithm is able to achieve global convergence for all the image voxels by using relative difference penalty and block sequential regularized expectation maximization approaches,^15^, ^16^, ^17^ incorporating point‐spread‐function (PSF) modelling.^18^, ^19^ The noise of the PET image is limited by a regularization parameter “beta value,” which is the only user‐input variable. Many studies demonstrated the advantages of the BPL reconstruction algorithm for evaluating small pulmonary nodules,^20^, ^21^ liver metastasis,^22^ and mediastinal nodes in nonsmall cell lung cancer.^23^

Teoh et al. examined beta values of 100–1000 on a nondigital PET/CT system with attenuation correction and scatter correction performed, and recommended a beta value of 400 for the optimal clinical use.^22^ They also used the beta value of 400 to assess small pulmonary nodules^20^ and mediastinal lymph nodes.^23^ Other researches applied fixed beta for BPL‐based reconstruction, for instance, a beta of 50 was used by Hsu et al. to characterize this new digital SiPM‐based PET/CT platform compared with other multimodal systems^24^; Howard et al. suggested a beta of 150 to observe small pulmonary nodules with improved visual conspicuity and SUVmax.^21^ All of the above studies have found that the increase in beta value would lead to the drop of SUV values (SUVmean and SUVmax) and elevation of SNR. However, none of them mentioned the size of lesions involved in their study and whether PVC was conducted or not. In addition, they have not measured the effect of beta value parameterization on the SUVpeak and contrast‐to‐noise ratio (CNR) in their clinical studies. The accuracy of SUVpeak likely to be less dependent on the image noise and lesion delineation.^10^ By defining SUVpeak, a fixed volume of interest (VOI, 1mL) is moved iteratively over the tumor to find the focus region with the highest mean value.^25^ The aim of this study is to evaluate the quantification accuracy and image quality with PVC and varied beta value of BPL‐based reconstruction as well as to optimize the SUV parameter for clinical applications of small pulmonary nodules.

## 2 MATERIALS AND METHODS

### 2.A Phantom study

#### 2.A.1 Phantom preparation

A National Electrical Manufacturers Association (NEMA) image quality phantom was used in the phantom study. The phantom has six spheres, with a diameter of 10, 13, 17, 22, 28, and 37 mm, respectively. Spheres were filled with 13.2 kBq/mL Fluoride ions in a 4‐to‐1 sphere to background activity ratio. Recovery coefficient (RC), contrast recovery (CR), and background variability (BV) were calculated using the equations as below [Eq. ((1), (2))–(3)].

(1) $$RC=\frac{A_{M}}{A_{K}}\times 100\%$$

(2) $$CR=\frac{\frac{A_{M}}{A_{B}}‐1}{C‐1}\times 100\%$$

(3) $$BV=\frac{SD_{B}}{C_{B}}\times 100\%$$

where *A_M_* is the measured mean activity concentration(in kBq/mL) within a VOI in each sphere delineated on CT images, A*_B_* is the measured mean activity concentration in the background, *A_K_* is the known activity concentration (in kBq/mL) in the sphere; *C* is the ratio of known activity concentration in the sphere and that in the background (that is four in the study), *SD_B_* is the standard deviation of the measured background activity concentration and *C_B_* is the mean of the corresponding measured background activity concentration.

#### 2.A.2 Phantom PET/CT imaging protocol

A SiPM‐based digital PET/CT system (Discovery MI, GE Healthcare, Milwaukee) with a 25 cm axial field‐of‐view was used to perform acquisitions. The phantom PET images were acquired with a bed position in List‐mode (3 min/bed position). The acquisition of CT images was followed at a voltage of 120 kVp, a tube current of 60–150 mA for SmartmA (mA modulation in the XY‐direction) and AutomA (mA modulation in the Z‐direction), a noise index (a parameter represents the desired noise level at the center of patient images for a given protocol) of 18, a pitch of 0.984 and a rotation time of 0.5 s. The phantom scans were performed three times to evaluate the variations and results are presented as means ± standard deviations (SD).

#### 2.A.3 Phantom image reconstruction

Both attenuation and scatter corrections were performed and the matrix size of reconstructed images was 256 × 256 with a pixel size (mm) of 2.73 × 2.73. A total of 345 slices was obtained for each scan with a slice thickness of 2.8 mm. All the PET images were reconstructed using the BPL‐based algorithm with a range of beta value (β) from 100 to 1000 in an interval of 50, where beta is defined in the BPL objective function as follow [Eq. (4)]:

(4) $$\hat{x}=\arg max_{x\geq 0}\sum y_{i}\text{log}{\left[P_{x}\right]}_{i}‐{\left[P_{x}\right]}_{i}‐\beta R\left(x\right)$$

where *x* is the image estimate, *i* is the pixel index, *y_i_* represents the measured PET coincidence data, *P* is the system geometry matrix, β is a regularization parameter, and R(x) is the relative difference penalty (RDP) to control noise.^15^ The RDP can be expanded as Eq. (5) shown below:

(5) $$R\left(\underline{x}\right)=\sum_{j=1}^{n_{v}}\sum_{k\in N_{j}}w_{j}w_{k}\frac{{\left(x_{j}‐x_{k}\right)}^{2}}{\left(x_{j}+x_{k}\right)+\gamma \left|x_{j}‐x_{k}\right|}$$

where γ is the parameter controls the edge preservation, w_j_ and w_k_ are relative weights for different components of the function and N_j_ represents a set of voxels surrounding voxel j. By applying the RDP to the objective function in Eq. (4), the reconstruction algorithm has the advantage of providing activity‐dependent noise control.^26^ With the increase of γ, images with sharper edges will be generated, which results in a more accurate quantification, especially for small lesions.^27^ PET Volume Viewer (GE Healthcare, Milwaukee) was employed to delineate the VOIs with a threshold of 42% (SUVmax) on the image with a beta value of 350 and then applied onto other images with different beta values. The calculation of SUVs was based on the equation shown below:

(6) $$SUV_{\mathrm{bw}}=\frac{r}{\left(\frac{a^{\prime }}{w}\right)}$$

where r is the activity concentration (kBq/ml), $a^{\prime }$ is the decay‐corrected dose of injected FDG (kBq), w is the body weight of the patient (g).^28^ The background region was selected according to NEMA NU2‐2012 guidelines.^29^, ^30^ For each sphere, different background regions were selected according to the sphere's diameter. As an example, the background activity concentration for the 37mm sphere was derived from 12 circular ROIs with a diameter of 37 mm were drawn. These 12 ROIs were located at background regions that did not contain any hot sphere and they were not allowed to overlap. The same set of 12 circular ROIs was then drawn on two slices above and two slices below the maximum intensity pixel to obtain a final of 60 background ROIs. The final background SUV reading was taken from the mean value of these 60 ROIs.^9^

### 2.B Clinical study

#### 2.B.1 Clinical characteristics

Patient informed consent is waived for the retrospective nature of this study. The ^18^F‐FDG PET/CT imaging data of the 19 consecutive patients from June 2018 to October 2018 was retrospectively analyzed. The patients consist of ten males and nine females; the median age of 67 yr old (range 55–84 yr old); the median height of 168 cm (range 155–180 cm); the median weight of 60.5 kg (range 47.5–80 kg).

A total of 53 nodules were found in these patients. Nodules located close to pleura and heart were excluded from this study due to the high Fluoro‐Deoxy‐Glucose (FDG) uptake of surrounding organ and tissue. In the end, 23 small pulmonary nodules were enrolled, and their diameter was ≤30 mm and the median diameter of pulmonary nodules was 15 mm (range 5.7–29.4 mm) measured on CT images. Lesions were categorized into two groups according to their size — the sub‐centimeter group (<10 mm, n = 7) and the medium‐size group (10–30 mm, n = 16).

#### 2.B.2 Patient preparation and PET/CT imaging protocol

The patients with glucose level under 200 mg/dl, received a dose of 2.96–3.7 MBq/kg ^18^F‐FDG after fasting 6 h, and then rested for approximately 1 h before scanning. The same PET/CT imaging protocol of the phantom study was used in the clinical study, but only scanned once.

#### 2.B.3 Clinical image reconstruction

Same to the phantom study, images were reconstructed using BPL (with the same beta value range and interval) together with TOF and PSF technologies. The attenuation correction and scatter correction were also performed.

#### 2.B.4 Qualitative imaging analysis

PET/CT images were visually evaluated by two readers (Dr. M. Liang and Dr. X.Z. Hao, with 5 and 10 yr of experience in nuclear medicine, respectively). Readers were blinded to any clinical information and the reconstruction method used. All scans were reviewed independently on a dedicated workstation (Advantage Workstation, Version 4.7; GE Healthcare) and in random order. Images reconstructed using different beta values were rated according to three image quality parameters: general image quality, image sharpness, and lesion conspicuity. The range of scores was from 1 to 5, where the score value of 5 represents the best performance, and noninteger score values were allowed. Scores given on the three image quality parameters of each beta value were summed up to give an overall image quality score.

#### 2.B.5 Quantitative imaging analysis

The SUVs of each primary lung tumor were recorded using a standard volume of interest (VOI, segmented by using a 42% threshold of the maximum SUV) tool.^31^ Moreover, background SUVs were assessed in the right lobe of the liver with 1.0‐cm‐diameter spherical VOIs. The SUVmean and the standard deviation of the standardized uptake value (SUVSD) within the VOIs recorded in the background for all reconstructions. The liver (17 patients) and descending aorta (2 patients) SUVSD was used as a measure of noise and the lesion's signal‐to‐noise ratio (SNR) was defined as the lesions SUVmax divided by the liver SUVSD.^32^ The reason for having two patients' measurements on descending aorta was too many metastatic lesions found on their liver. Moreover, measurements on the contrast‐to‐noise ratio (CNR) were conducted to evaluate the lesion detectability. The equation for CNR can be found below in Eq. (7)_:_ ^33^

(7) $$\text{CNR}=\frac{Mean\left(\mathrm{lesion}\right)‐Mean\left(\mathrm{background}\right)}{SD\left(\mathrm{background}\right)}$$

Unlike SNR, the background region of CNR was defined in the lesion's neighboring lung tissue. The Mean(lesion) and Mean(background) represent the mean SUV value of the lesion or background in the mean image and SD (background) is the standard deviation value of the background.

#### 2.B.6 PVC for pulmonary nodules

The PVC method used in this study was a simplified version modified from Kumar et al.^34^ With the results from the phantom study, the relationships between RC and sphere diameter reconstructed using different beta values were established using linear regression analysis. The purpose of generating this linear regression model from the phantom study is to predict the RC values for the PVC of the SUVs of pulmonary modules of similar diameter and contrast in clinical data. The diameter of each nodule was measured on CT images, which was then input into its corresponded linear regression function to calculate its estimated RC. Each nodule's quantification parameters (SUVmean, SUVmax, and SUVpeak) were then divided by its estimated RC to achieve the PVC. This simplified method assumed that the tumor‐to‐background ratio for all pulmonary nodules was 4:1. The reason for not having more phantom linear regression models under different contrast was that the current study focused on the small pulmonary nodules and their TBR values were close to 4:1.

#### 2.B.7 Statistical analysis

Statistical analysis was conducted using SPSS Statistics 22.0 (IBM Co., New York, USA). All data are displayed as mean ± SD, except from the subjective image analysis scores which are displayed as median numbers. Paired t‐test was used to compare the difference in SNR between beta of 100 and 1000. Pearson correlation test was performed to evaluate the relationship between SUVs and beta values. Cohen’s kappa coefficient (κ) was used to evaluate the inter‐rate agreement. *P* < 0.05 is considered as a statistically significant difference, while *P* < 0.001 is taken as a highly significant difference.

## 3 RESULTS

### 3.A Phantom studies

**FIG. 1:**
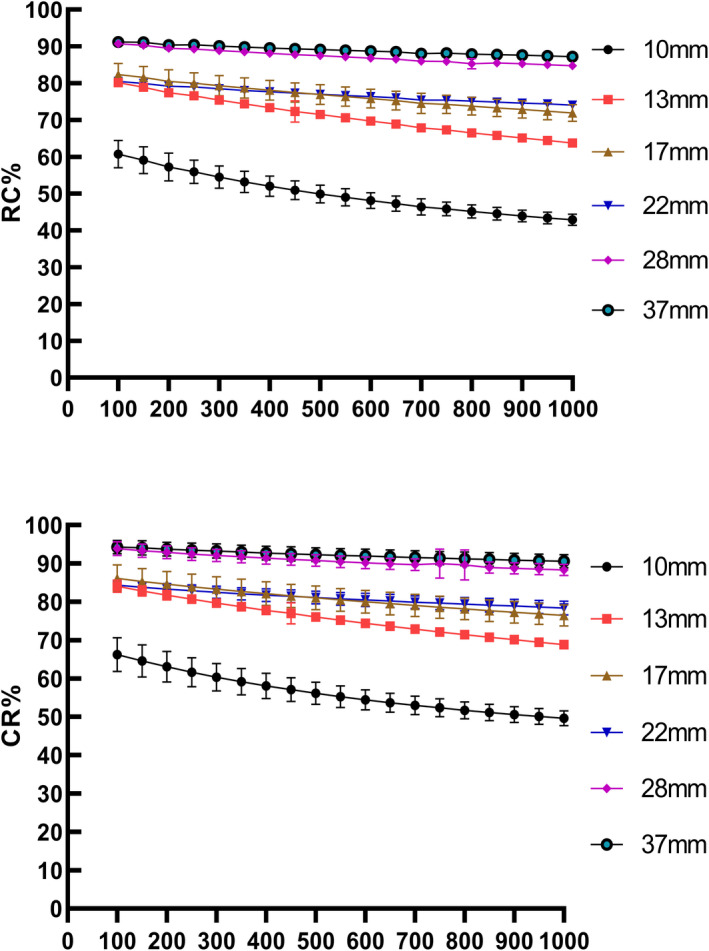
Comparison of contrast recovery (above) and recovery coefficient (below) among positron emission tomography reconstruction methods for six hot spheres (diameter 10–37 mm) filled with 13.2 kBq/mL Fluoride ions in a 4‐to‐1 contrast ratio.

**FIG. 2:**
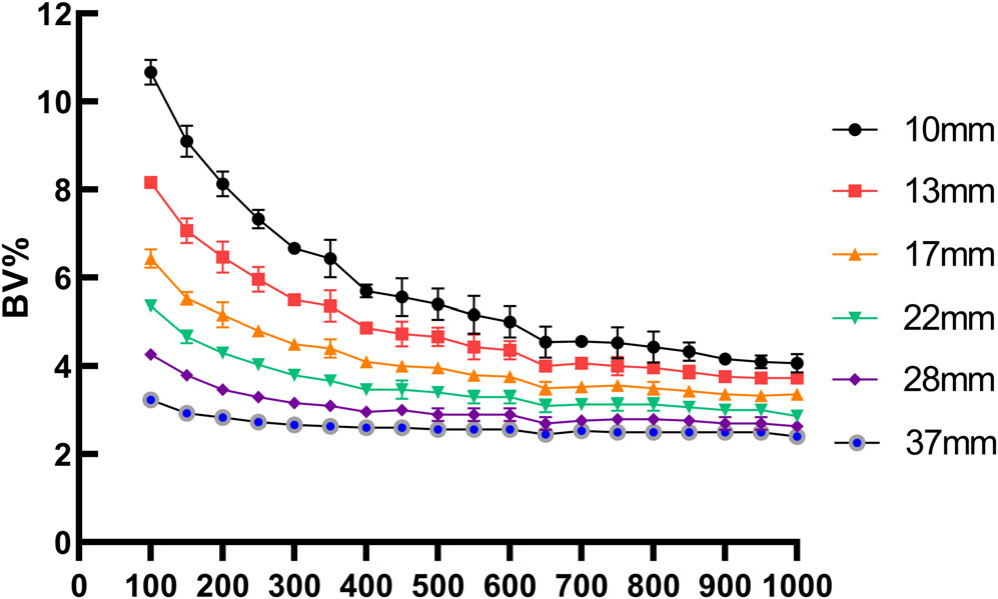
Comparison of background variability among positron emission tomography reconstruction methods for six hot spheres (diameter 10–37 mm) filled with 13.2 kBq/mL Fluoride ions in a 4‐to‐1 contrast ratio.

**FIG. 3:**
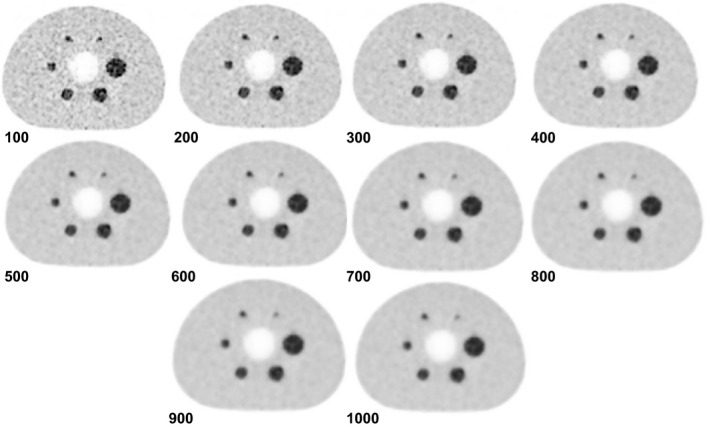
Comparison of different beta values (numbers next to each image) of National Electrical Manufacturers Association phantom reconstructed using the Bayesian penalized likelihood algorithm.

The results for the phantom study are illustrated in Figs. 1, 2, 3. As beta value increased, the CR, RC, and BV decreased for all spheres. By considering the size of different spheres throughout the whole beta value range, both CR and RC values (Fig. 1) increased with the sphere's diameter. In particular, small spheres (10 and 13 mm) were observed having steeper gradient as beta value went up compared with large spheres. Curves for BV values of all spheres (Fig. 2) dropped to a plateau when the beta value was higher than 650 and the figure also showed that large size spheres gave lower BV values compared with small spheres.

### 3.B Clinical studies

**FIG. 4:**
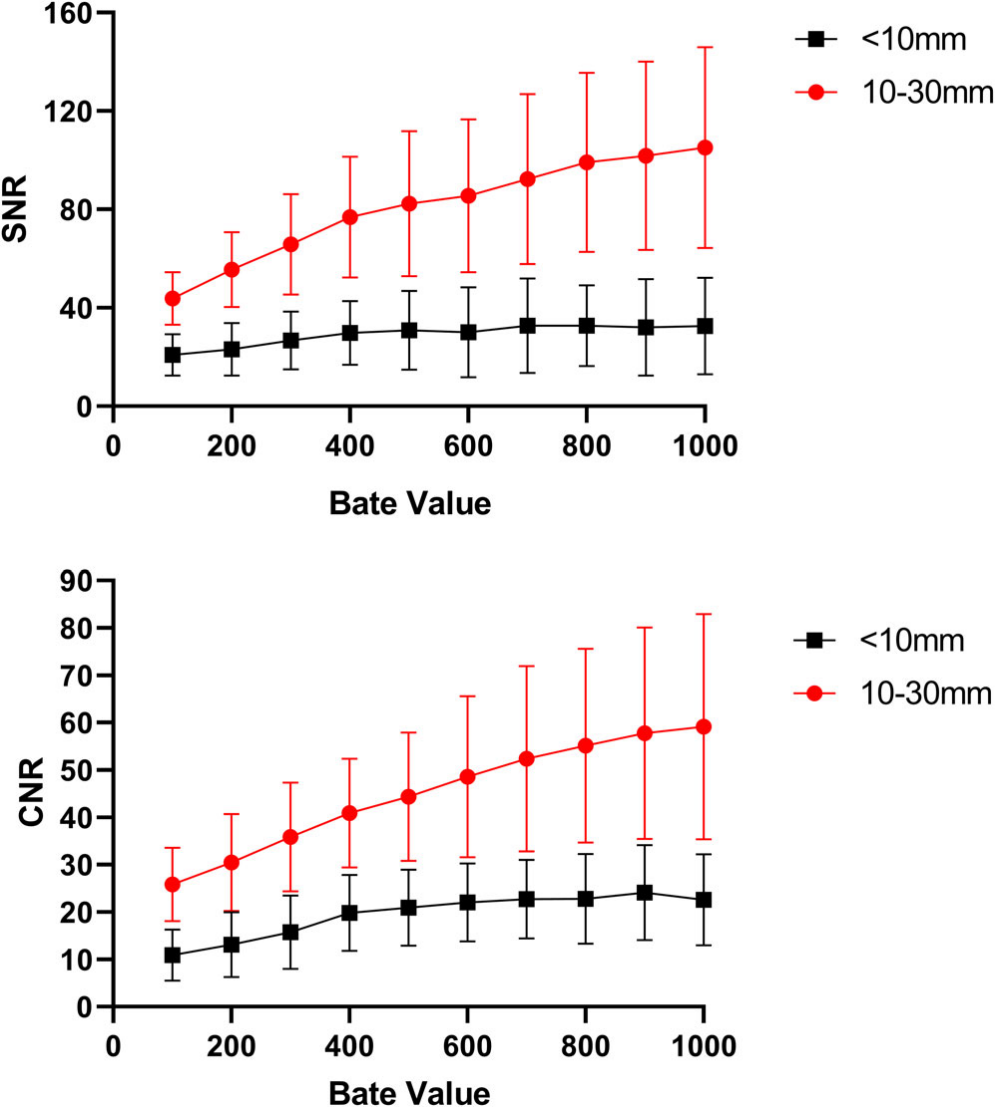
**(**a) The Signal‐to‐noise ratio (SNR) of 23 small pulmonary nodules measured using a various range of beta values. (b) The values of contrast‐to‐noise ratio (CNR) of the same small pulmonary nodules. Highly significant differences in SNR and CNR were found between those measured at beta = 100 to beta = 1000 for both groups (*P* < 0.001, for all).

SNR of 23 lesions is displayed as mean values with SD in the Fig. 4(a). Steady increases in SNR with beta values were found for both groups from beta value of 100 to 700. When beta values were higher than 700, SNR of the 10–30 mm group continued to increase while that of the <10 mm group reached a plateau and the overall increment was only 3.05 ± 4.86. The statistical analysis shows that both groups have highly significant difference in SNR from beta of 100 to 1000. By conducting the measurements on CNR, similar trends to SNR were observed for both size groups among beta values [Fig. 4(b)].

**FIG. 5:**
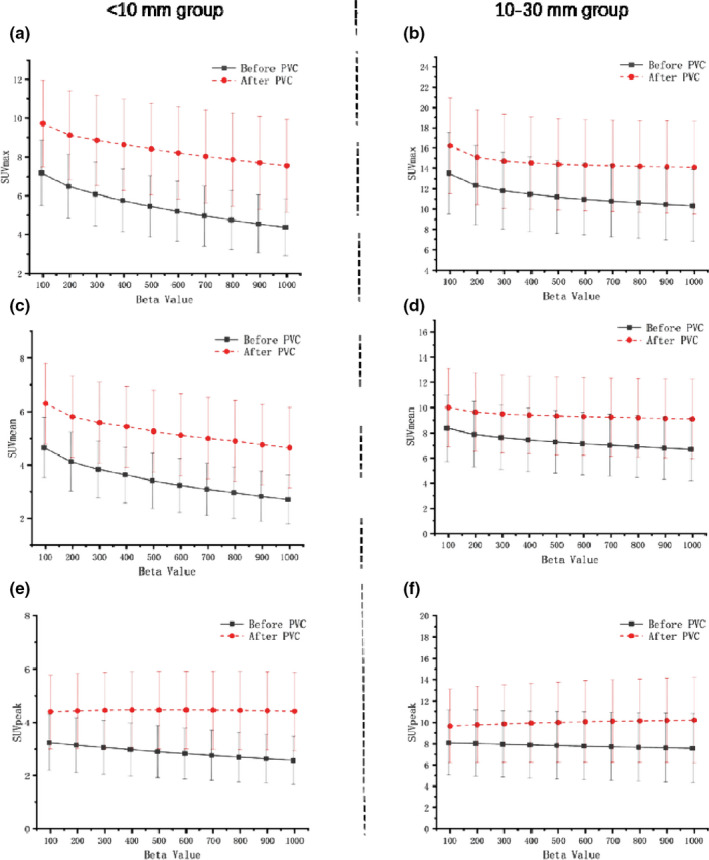
Standardized uptake values (SUVs) of two size groups measured using a various range of beta values. The sub‐centimeter group (a, c, and e) showed highly significant negative correlations of SUVs with beta values before PVC (*P* < 0.001), but SUVpeak showed no significant difference (*P* = 0.830) after the PVC. The median size group (f) showed a highly significant positive correlation of SUVpeak across the beta values after PVC (*P* < 0.001), although the overall changes were considerably small (0.57 ± 0.58).

By using the phantom‐derived RC linear regression model with a contrast of 4:1, PVE‐corrected SUVs of pulmonary nodules were obtained. The impact of increasing beta values on SUVs estimated before and after the PVC is illustrated in Fig. 5. Before the PVC was performed, highly significant negative correlations were observed between all SUVs and beta values for both size groups (*P* < 0.001 for all). After the PVC was performed, SUVmean and SUVmax of both groups were found having highly significant negative correlations (*P* < 0.001 for all). However, SUVpeak of the <10 mm group was found no significantly different (*P* > 0.05) among all different beta values examined. In contrast, SUVpeak of the 10–30 mm group was determined to have a highly significant positive correlation with beta value (*P* < 0.001), although the overall SUVpeak change between beta = 100 and beta = 1000 was only 0.57 ± 0.58.

### 3.C Subjective image quality evaluation

The results of the subjective image assessment including all study subjects are given in Table 1. For both size groups, score given from the two readers for general image quality, image sharpness, and lesion conspicuity demonstrated certain agreement (κ = 0.688 for general image quality, 0.864 for image sharpness, 0.798 for lesion conspicuity, and 0.765 for overall score). From both readers, the beta value of 300 had the highest overall score for the <10 mm group, and 400 was rated the highest overall score for the 10–30 mm group.

**TABLE 1:** Results of subjective positron emission tomography (PET) image quality ratings for different beta value.

Beta value	General image quality		Image sharpness		Lesion conspicuity		Overall score	
	Reader 1	Reader 2	Reader 1	Reader 2	Reader 1	Reader 2	Reader 1	Reader 2
<10 mm group								
100	2	2	2	2	2	2	6	6
200	3	3	2	3	3	3	8	9
300	5	4	4	4	4	4	13	12
400	4	4	3	3	4	4	11	11
500	3	3	3	3	3	3	9	9
600	2	2	3	3	2	2	7	7
700	1	1	2	2	1	1	4	4
800	1	1	1	1	1	1	3	3
900	1	1	1	1	1	1	3	3
1000	1	1	1	1	1	1	3	3
10‐30 mm group								
100	1	1	2	2	1.5	1.5	4.5	4.5
200	2	2.5	2	2	2	2	6	6.5
300	3	3	3.5	3.5	3	4	9.5	10.5
400	4	4	4	4	4	4	12	12
500	3	3	3	3	3	3	9	9
600	2	2	2.5	2.5	2	2	6.5	6.5
700	2	1.5	2	2	2	1	6	4.5
800	1	1	1	1	1	1	3	3
900	1	1	1	1	1	1	3	3
1000	1	1	1	1	1	1	3	3

The framed columns are the reconstructed datasets yielding the highest score for each assessed parameter. Values are displayed as median numbers.

## 4 DISCUSSION

This study has investigated the relationship between beta values and quantification accuracy, as well as the image quality. In contrast to previous studies, we also performed PVC and measured SUVpeak and CNR to investigate in more detail the effect of beta values.

In phantom studies, the RC and CR values reflect the quantification accuracy of PET. Results from our study showed that RC and CR values decreased while beta value went up which suggested that the selection of a high beta value may have a negative effect on SUV estimation accuracy. The BV value, on the other hand, is one of the parameters representing the image noise level. BV was found lower in reconstructed images with high beta values for all spheres which implicated a lower level of noise was achieved by using a higher beta value and this was expected as the beta value is known as the noise penalization factor in the BPL algorithm. Our phantom results are consistent with literature results, reported by Teoh et al.^23^ and Linstrom et al.^35^ On top of this, we also reported the effect of beta values on RC values. By using the relationship between RCs and the sphere diameter, we established a phantom‐derived linear regression model at the contrast of 4:1 to estimate the clinical RC and to perform PVC.

In clinical studies, SNR results showed that noise level decreased with high beta values which indicated that images reconstructed with a high beta value might have better quality compared to those using low beta values. However, unlike the medium‐size group, SNR of the <10 mm group showed that such noise reduction became less obvious when beta values reached over 700, which agrees with our phantom study where BV reached a plateau when beta value was 650. In addition, the SNR values for small lesions (<10 mm) were consistently lower than the medium‐size group (10–30 mm) and this was caused by the PVE, which affected more on small lesions. The effect of beta on SNR was also reported by Linstrom et al.^36^ and their results were similar to this study although the subjects they enrolled were mixed with different diseases and they did not categorize lesions into different sizes. The clinical result of SNR suggested that the increase in the regularization parameter can reduce the image noise, but for small lesions, such improvement will become less obvious. The results for CNR agreed with SNR, the increase in beta value led to a higher CNR and this was contributed by the reduction in noise. At this stage, CNR results suggest that a better lesion detectability could be achieved by having a high beta value.

In the clinical assessment of ^18^F‐FDG PET/CT quantification, we applied PVC on all pulmonary nodules as the partial volume effect has a great impact on the quantification accuracy of small lesions. Before the PVC, regardless of nodule sizes, the highly significant negative correlation found between beta values and SUVs implied that a choice of very high beta value will lead to the loss of quantification accuracy. Howard et al. reported that, for small pulmonary nodules, a decreasing trend was observed in SUVmax when beta value increased from 150 to 350 (*P* < 0.01), although they did not state whether PVC was performed and no further information on SUVmean, SUVpeak, and lesion sizes was found.^21^

After PVC was performed, SUVmean and SUVmax of both groups showed negative correlations. Notably, such negative correlation was not found between beta value and SUVpeak measured from the <10 mm group after the PVC [Fig. 4(e) red]. This suggests that the drawback of increasing beta values on quantification accuracy may not be significant for sub‐centimeter lesions when PVC is performed and SUVpeak is chosen as the quantification parameter. For the medium‐size group, correlations between SUVpeak and beta values changed to a positive correlation after PVC [Fig. 4(f) red] even though the overall change was only 0.57 ± 0.58. The small variations found in SUVpeak across different beta values can be explained by recalling the definitions of different SUV parameters. SUVmean is highly dependent on the lesion delineation as it measures the uptake of ROI around the maximum pixel, and a change in beta value may affect the ROI definition which consequently affects the quantification accuracy.^35^ In addition, SUVmax represents uptake in the highest metabolic region which has the maximum pixel value, and it tends to be affected by the different noise levels.^37^ In contrast, SUVpeak was introduced in the PET Response Criteria in Solid Tumors (PERCIST) framework in 2009. SUVpeak is defined in a fixed volume of interest (VOI, 1mL) with the highest mean value. This focus region does not always have the highest maximum SUV value and does not even contain the pixel with the maximum SUV in some cases.^38^ As a result, SUVpeak is likely to be less dependent on image noise and lesion delineation.^10^ Vanderhoek et al. evaluated the treatment response using PET‐based quantification and compared the difference given from different SUV parameters. It was found that SUVmean and SUVtotal yielded the largest difference (up to 90%) while the variations between SUVpeak and SUVmax stayed as the smallest.^39^ A study on finding the variability given from different SUV parameters was conducted by Brendle et al. and they stated SUVpeak as the most reliable method for small lesions particularly.^36^ In agreement with our clinical results, the use of PVC‐corrected SUVpeak as a quantification metric is recommended to effectively avoid the loss of quantification accuracy that may be caused by the choice of high beta values, especially for sub‐centimeter pulmonary nodules. Scores given from the subjective image quality assessment showed there was a different beta value preference for the different lesion sizes (300 for the <10 mm group and 400 for the 10–30 mm group). Interestingly, although both SNR results from the phantom and clinical studies showed that high beta values could provide lower image noise, physicians rated the image quality, image sharpness, and lesion conspicuity as the lowest score (1 point) for images reconstructed using beta values higher than 800. Moreover, while the quantitative results of CNR suggested that a higher beta value might result in a better lesion detectability, the “lesion conspicuity” score demonstrated a different result. The difference in results given from the quantitative image quality analysis and subjective image quality analysis confirmed that image noise level is not the only feature affecting the overall image quality.

It is important to point out that the optimal beta values suggested by physicians in this study are only for small pulmonary nodules using ^18^F‐FDG PET/CT. As reported by Teoh et al., beta values of 200 and 300 were recommended for recurrent prostate cancer using ^18^F‐fluciclovine PET/CT scan.^40^ Rowley et al. even suggested a beta value of 4000 as an optimal option for imaging using ^90^Y‐SIRT PET/CT scan.^41^ As a result, it is still a challenge to identify a universal value for the noise penalization factor, although most of FDG‐based PET/CT studies suggested low beta values for most applications. Notably, Yamaguchi et al. reported that, from the phantom study on BPL reconstruction, the activity concentration line profile for the small sphere (10 mm) appeared having an overshoot when the sphere to background ratio was too high (16:1) but this overshoot can be compensated by using higher beta values.^42^

One of the major limitations of this study is the accuracy of the PVC method. Correction factors were estimated from the NEMA phantom study for which hot spheres in the uniform background were considered. In reality, nodules could be surrounded by a region of high uptake. In addition, spill‐in of activity from large surrounding structures will most likely occur. For such cases, the proposed PVC will lead to inaccuracy and misinterpretation (overestimation of activity and false positive). In order to avoid such scenario, patients with nodules located close to the heart or pleura were not considered. In addition, this study assumed that the tumor‐to‐background ratio was approximately 4:1 for all nodules when applying the PVC. Although this contrast ratio was close for those small pulmonary nodules, PVC models with more comprehensive contrasts are needed in the future study to achieve better accuracy. Another challenge with the proposed PVC is that the lesion in this study was defined on the CT images, which might not correspond to that on the PET image due to motion between/during scans. Normally, the solution was to apply the respiratory gating technology. However, respiratory gating was not widely used in our hospital by considering the convenience. Moreover, the smallest sphere diameter of the NEMA phantom used in this study was 10 mm. This means the phantom‐derived PVC model did not cover small lesions with the diameter smaller than 10 mm, and accuracy of PVE‐corrected uptake values for those small lesions might be affected. Patients enrolled in this study were not histopathologically confirmed, which prevented our study from investigating the impact of beta values on benign and malignant tumors. Finally, the number of patients enrolled was considerably low compared to past studies. Future studies should employ a more appropriate PVC method considering any potential spill‐in effects to ensure more accurate quantification for a wider range of lesions. Moreover, the effects of beta parameter values on a larger set of independent factors should be investigated, including patient body weight, body mass index, different histopathology, and different radiotracers to give clearer guidance for choosing appropriate beta values on clinical applications.

## 5 CONCLUSION

Our results suggest that on a novel SiPM PET/CT using the BPL algorithm, increasing the beta value can effectively reduce the image noise. More importantly, it is recommended to perform PVC and choose SUVpeak as the quantification parameter to mitigate the effects of beta regularization, especially for pulmonary nodules with a diameter smaller than 10 mm. In addition, beta values of 300 and 400 were recommended by observers for the sub‐centimeter group and the median size group, respectively.

## CONFLICT OF INTEREST

The authors declare no conflict of interest.

## AUTHORS CONTRIBUTIONS

Z.W carried out the research methods, participated in design of this study, and drafted the manuscript. B. G, B. H, B. Z, and Z. Q reconstructed the PET images with different bate values and calculated related parameters. M. L and X. H scored the image quality and performed the statistical analysis. J. X and S. L conceived of the study, and participated in its design and coordination. All authors read and approved the final manuscript.
